# Plasmid Classification in an Era of Whole-Genome Sequencing: Application in Studies of Antibiotic Resistance Epidemiology

**DOI:** 10.3389/fmicb.2017.00182

**Published:** 2017-02-09

**Authors:** Alex Orlek, Nicole Stoesser, Muna F. Anjum, Michel Doumith, Matthew J. Ellington, Tim Peto, Derrick Crook, Neil Woodford, A. Sarah Walker, Hang Phan, Anna E. Sheppard

**Affiliations:** ^1^Nuffield Department of Medicine, John Radcliffe Hospital, University of OxfordOxford, UK; ^2^National Institute for Health Research Health Protection Research Unit in Healthcare Associated Infections and Antimicrobial Resistance, University of OxfordOxford, UK; ^3^Department of Bacteriology, Animal and Plant Health AgencyAddlestone, UK; ^4^Antimicrobial Resistance and Healthcare Associated Infections Reference Unit, Public Health EnglandLondon, UK

**Keywords:** plasmid typing, whole-genome sequencing, antibiotic resistance, genomic epidemiology, network analysis

## Abstract

Plasmids are extra-chromosomal genetic elements ubiquitous in bacteria, and commonly transmissible between host cells. Their genomes include variable repertoires of ‘accessory genes,’ such as antibiotic resistance genes, as well as ‘backbone’ loci which are largely conserved within plasmid families, and often involved in key plasmid-specific functions (e.g., replication, stable inheritance, mobility). Classifying plasmids into different types according to their phylogenetic relatedness provides insight into the epidemiology of plasmid-mediated antibiotic resistance. Current typing schemes exploit backbone loci associated with replication (replicon typing), or plasmid mobility (MOB typing). Conventional PCR-based methods for plasmid typing remain widely used. With the emergence of whole-genome sequencing (WGS), large datasets can be analyzed using *in silico* plasmid typing methods. However, short reads from popular high-throughput sequencers can be challenging to assemble, so complete plasmid sequences may not be accurately reconstructed. Therefore, localizing resistance genes to specific plasmids may be difficult, limiting epidemiological insight. Long-read sequencing will become increasingly popular as costs decline, especially when resolving accurate plasmid structures is the primary goal. This review discusses the application of plasmid classification in WGS-based studies of antibiotic resistance epidemiology; novel *in silico* plasmid analysis tools are highlighted. Due to the diverse and plastic nature of plasmid genomes, current typing schemes do not classify all plasmids, and identifying conserved, phylogenetically concordant genes for subtyping and phylogenetics is challenging. Analyzing plasmids as nodes in a network that represents gene-sharing relationships between plasmids provides a complementary way to assess plasmid diversity, and allows inferences about horizontal gene transfer to be made.

## Introduction

Plasmid genomes generally include a ‘backbone’ of core genetic loci, which are somewhat conserved amongst broadly related plasmids of the same family (Phan et al., 2009), and associated with key plasmid-specific functions such as replication and mobility. Accessory genes may also be present, and often confer clinically relevant traits such as virulence and antibiotic resistance (Thomas and Summers, 2008). Plasmids can act as efficient vectors of horizontal gene transfer (HGT). Notably, during conjugation, a plasmid promotes its own transfer (and/or that of a co-resident plasmid) from one bacterial cell to another (Norman et al., 2009). Accessory genes are therefore frequently spread by virtue of being located on transmissible plasmids; moreover, they are commonly associated with smaller mobile elements such as transposons, facilitating intracellular mobilization amongst plasmids, or to the chromosome (Stokes and Gillings, 2011). Due to their ability to transmit genes encoding adaptive traits across bacterial populations, plasmids can enable bacteria to evolve rapidly under environmental pressure (Heuer and Smalla, 2012). A striking example of bacterial adaptive evolution is that of antibiotic resistance, which is driven, in part, by dissemination of resistance plasmids (plasmids conferring antibiotic resistance), and now threatens modern medicine (Carattoli, 2013; World Health Organization, 2014).

Classifying plasmids according to a typing scheme provides useful insights into the epidemiology of plasmid-mediated antibiotic resistance: for example, studying the composition of plasmid types can indicate whether an antibiotic resistance epidemic is driven by diverse plasmids or one dominant plasmid type (Valverde et al., 2009). In addition, hypotheses about resistance transmission during outbreaks can be refined according to the relatedness of resistance plasmids harbored by clinical strains (Pecora et al., 2015). The principal plasmid classification schemes are replicon and MOB typing, based on backbone loci encoding plasmid replication and mobility functions, respectively (Carattoli et al., 2005; Garcillán-Barcia et al., 2009). Whilst these single-locus typing schemes have been widely and successfully applied, they provide limited resolution (Fricke et al., 2009), restricting epidemiological inference: in an outbreak context, if two patients are infected by unrelated strains harboring resistance plasmids of the same type, this raises the possibility of plasmid transmission, but plasmid transmission cannot be conclusively ruled-in using single-locus plasmid typing alone; further higher-resolution investigation would be required (Foxman et al., 2005). If resistance plasmids are unrelated, plasmid transmission can be ruled-out, though a transmission link via resistance gene transposition is possible.

Plasmid typing may provide a stepping-stone to higher resolution analyses; identifying shared (‘core’) genes amongst related plasmids can inform development of plasmid multi-locus sequence typing (pMLST) schemes (García-Fernández et al., 2011), or allow phylogenetic relationships to be reconstructed based on core gene single nucleotide polymorphisms (SNPs) (de Been et al., 2014). Unfortunately, determining high-resolution plasmid relationships is challenging: the tendency of plasmids to gain, lose and rearrange genetic content means sets of plasmids – even if of the same type – will tend to share few phylogenetically concordant core genes (Fondi et al., 2010; Tazzyman and Bonhoeffer, 2014), impeding subtyping and phylogenetic analysis (Maiden, 2006). Even backbone genes may not be well conserved across all plasmids of the same type (Lanza et al., 2014), and sometimes show mosaic phylogenetic origins (Sen et al., 2013).

Whole-genome sequencing (WGS) data can now be obtained for many bacterial isolates, at relatively low cost, within short timescales (Metzker, 2010). Whilst sequencing reads from a bacterial isolate represent plasmid(s) as well as the chromosome, WGS-based studies have often focused on the host strain chromosome as the unit of interest (Croucher and Didelot, 2015). For strain-level clinical surveillance to elucidate antibiotic resistance transmission routes, dissemination should primarily involve clonal transmission of particular antibiotic-resistant strains. However, recent analyses indicate that plasmids may transmit between strains frequently, even over short timescales (Conlan et al., 2014; Sheppard et al., 2016). Therefore, the chain of transmission no longer simply corresponds to strain transmission; resistance plasmid dissemination across strains recruits different recipient strains into the outbreak too, resulting in a ‘plasmid outbreak.’ Although insight may be limited by difficulties in determining high-resolution plasmid relationships, these dynamics mean that plasmid analysis across a variety of strains is important, including for short-term surveillance studies (Adler and Carmeli, 2011).

Conventional PCR-based plasmid typing methods are commonly used, but *in silico* approaches for classifying sequenced plasmids are also available. WGS datasets from short-read sequencing projects offer exciting opportunities for large-scale plasmid analysis, while presenting the additional challenge of assembling reads to resolve individual plasmid structures. After summarizing current plasmid classification schemes (replicon and MOB typing), this review discusses the opportunities and challenges of conducting *in silico* plasmid typing on WGS datasets to gain insight into plasmid-mediated resistance epidemiology. We highlight novel tools for WGS-based plasmid analysis, and examine gene-sharing networks as a complementary approach for analyzing plasmid relationships. This review focuses on WGS datasets from cultured rather than metagenomic samples; for the latter, see recent reviews (Jørgensen et al., 2014; Martínez et al., 2016).

## Plasmid Typing Schemes

Replicon typing schemes exploit genetic elements of the replicon region (encoding replication machinery) (**Table 1**). Couturier et al. (1988) typed plasmids according to Southern blot hybridization, using replicons from plasmids of different incompatibility groups as probes. However, this method is limited by probe cross-hybridization amongst closely related replicon sequences (Carattoli, 2009). PCR-based replicon typing (PBRT) – where plasmids are typed according to PCRs targeting various replicon sequences – is less laborious, and shows higher specificity in detecting replicons (Carattoli et al., 2005). For gram-negative bacteria, PBRT schemes targeting replicons found in Enterobacteriaceae and *Acinetobacter baumannii* plasmids are available (Carattoli et al., 2005; Bertini et al., 2010). A PBRT scheme for plasmids of gram-positive bacteria has been developed, focusing on enterococcal (Jensen et al., 2010) and staphylococcal (Lozano et al., 2012) plasmids. For common Enterobacteriaceae replicon types, pMLST schemes have been devised for subtyping (Brolund and Sandegren, 2016; Hancock et al., 2016). Availability of WGS data has motivated the development of *in silico* replicon typing and subtyping tools, which have been validated for Enterobacteriaceae plasmids (Carattoli et al., 2014). For plasmids from taxa not represented by existing *in silico* tools, *ad hoc in silico* methods have been derived from PBRT schemes (Shintani et al., 2015; Brodrick et al., 2016).

**Table 1:** Summary of plasmid typing and subtyping schemes.

Plasmid typing schemes		Comments
Replicon typing	Inc grouping	Plasmids with similar replication machinery are often unable to stably co-exist within the same host cell (Snyder et al., 2013); this phenomenon was traditionally used to classify plasmids into incompatibility (Inc) groups. Inc grouping has been applied to plasmids from Enterobacteriaceae (Hedges and Datta, 1973), *Pseudomonas aeruginosa*, and *Staphylococcus aureus* (Taylor et al., 2004).
	Replicon probe hybridization	Couturier et al. (1988) cloned replicons representing Enterobacteriaceae Inc groups; plasmids were classified according to Southern blot hybridizations using the replicons as probes. Probe hybridization lacks specificity when closely related replicons are present, and is no longer widely used except for its application subsequent to PCR-based replicon typing (PBRT); here, amplicons derived from PCR can be used as probes to type plasmids isolated on a gel (EFSA, 2011).
	PCR-based replicon typing (PBRT)	PBRT for plasmids of the well-studied Enterobacteriaceae family currently detects 28 replicons (based on various genetic loci including *rep* genes and replication regulatory sequences). These PBRT types roughly correspond to traditional Inc groups, so Inc nomenclature is still used. A commercial 28-replicon PBRT kit is available (Diatheva, 2016). More recently, PBRT has been devised for *Acinetobacter baumannii* plasmids (Bertini et al., 2010); multiplex PCRs targeting 27 replicons are used to classify plasmids into 19 ‘GR’ types. A PBRT scheme has also been applied to plasmids of gram-positive taxa, focusing on enterococcal (Jensen et al., 2010) and staphylococcal (Lozano et al., 2012) plasmids. A closely related scheme focuses on plasmids of *Enterococcus faecium* (Rosvoll et al., 2010, 2012).
	Replicon subtyping	Allelic profiles are assessed at 2–6 core loci (depending on the specific scheme). Plasmids are assigned a pMLST subtype nesting within the broader replicon type. pMLST schemes are available for six common replicon types of Enterobacteriaceae plasmids (IncF, HI1, HI2, I1, N, A/C). PCR-based and *in silico* methods are available.
	*In silico* replicon typing/subtyping	Replicon and pMLST allele databases can be downloaded for local use, but user-friendly web-tools (PlasmidFinder/pMLST for replicon typing/subtyping) can run the analysis pipeline, including read assembly. The PlasmidFinder replicon database currently contains 121 reference replicons for Enterobacteriaceae plasmids; a dataset of replicons for gram-positive plasmids based on the scheme devised by Jensen et al. (2010) and Lozano et al. (2012) is also available (Carattoli et al., 2014; Center for Genomic Epidemiology, 2016). Instead of relying on read assembly and BLAST, unassembled reads can be mapped to the PlasmidFinder database or pMLST database using SRST2 (Inouye et al., 2014).
MOB typing	PCR-based MOB typing	PCR-based ‘degenerate primer MOB typing’ (DPMT) is used to type γ-Proteobacterial plasmids; 19 degenerate primer pairs target relaxase sequences to partition plasmids into five of the main MOB types identified by *in silico* MOB typing (Rose et al., 1998; Alvarado et al., 2012). PCR-based MOB typing has also been demonstrated for enterococcal plasmids (Goicoechea et al., 2008; Freitas et al., 2016).
	*In silico* MOB typing	Six N-terminal relaxase sequences are used as PSI-BLAST probes to detect relaxase sequences of transmissible plasmids, and partition plasmids into six possible MOB types (Garcillán-Barcia et al., 2009).
Other locus-targeting schemes		Other locus-based methods for plasmid typing and subtyping exist, but tend to be applicable to a more restricted set of plasmids (Guglielmini et al., 2011; Freitas et al., 2013; Compain et al., 2014; Dealtry et al., 2014a,b; Bousquet et al., 2015).
Plasmid ‘fingerprinting’ (RFLP typing)		Restriction fragment length polymorphism (RFLP) is sometimes used to subtype plasmids, especially when pMLST is unavailable. However, band patterns can be difficult to interpret, and do not provide a reliable phylogenetic marker (Laguerre et al., 1992). Shearer et al. (2011) used RFLP to assign a subset of conserved staphylococcal plasmids to three major RFLP types.

MOB typing exploits the conserved N-terminal sequence of the relaxase proteins encoded by transmissible plasmids (Francia et al., 2004; Garcillán-Barcia et al., 2009). As with replicon typing, both PCR-based and *in silico* approaches are used for MOB typing (**Table 1**). Compared with replicon typing, MOB typing classifies plasmids at lower resolution (Garcillán-Barcia et al., 2011).

A drawback of replicon typing is that individual plasmids can contain multiple replicons, complicating classification, whereas usually just one relaxase is encoded. However, due to its finer resolution, replicon typing provides more detailed information on plasmid relatedness, particularly if a pMLST subtyping scheme is available (Garcillán-Barcia and de la Cruz, 2013). Even within relatively well-studied taxa, neither scheme classifies all plasmids, likely reflecting diversity in plasmid backbones. Shintani et al. (2015) assessed *in silico* typing, and found that the proportion of Enterobacteriaceae plasmids that could be replicon typed was 75%; for *Acinetobacter* plasmids the proportion was 67%. Only around half of plasmids from major gram-positive taxa could be replicon typed (51% Firmicutes plasmids, 49% Actinobacteria plasmids), although the proportion was higher for enterococcal and staphylococcal plasmids (83 and 85% respectively) (Supplementary Table S1 in Shintani et al., 2015). Lanza et al. (2015) also highlight gaps in replicon typing of Firmicutes plasmids. MOB typing only types transmissible plasmids (∼50% γ-Proteobacterial plasmids; ∼35% Firmicutes plasmids) (Smillie et al., 2010).

## WGS Data for Plasmid Classification: Opportunities and Challenges

When analyzing whole genomic DNA, limited information can be derived from plasmid typing alone: bacterial cells may contain multiple different plasmids, and a single plasmid may contain multiple replicons, obscuring correspondence between detected replicons and the set of plasmid types within a host cell (Johnson et al., 2007). Therefore, in PCR-based studies – where genomic context of an amplicon remains unknown – plasmids are commonly isolated first, before being individually characterized by replicon and resistance typing (see Table 11 in EFSA, 2011). This is time-consuming, restricts the number of isolates that can be analyzed, and has inherent limitations: if plasmids from the same isolate are of similar size they cannot be separated by pulsed-field gel electrophoresis, and isolation by transfer to recipient cells is not always achieved (Dib et al., 2015).

Potentially, WGS enables *in silico* analyses in which plasmid typing and analysis of loci of interest, such as resistance genes (and their plasmid or chromosomal genetic context), are performed in a unified way. Consequently, much larger isolate collections can be analyzed – a key advantage given that plasmid studies have indicated a need for including more strains in analyses (e.g., environmental strains, or isolates exhibiting only low-level resistance) to uncover more complex transmission routes (Carrër et al., 2010; Stoesser et al., 2014, 2015a). Furthermore, a thorough mechanistic understanding of clinically important aspects of plasmid biology, such as host range and phenotypic effects, may require analysis of the wider plasmid genome rather than specific loci.

Unfortunately, there are significant obstacles to *in silico* analysis of WGS data. Popular high-throughput sequencing technologies (e.g., Illumina) produce short (∼100–300 bp) reads, for which assembly is inherently challenging (Nagarajan and Pop, 2013). Isolating individual plasmids prior to sequencing simplifies assembly, potentially enabling complete plasmid reconstruction (Mathers et al., 2015), but is laborious. Long-read sequencing vastly simplifies assembly, but high costs restrict its use (Koren and Phillippy, 2015). Therefore, a major challenge lies in extracting useful information from short sequencing reads derived from different sources (different co-resident plasmids, the chromosome).

### Reference-Based Read Mapping versus *De novo* Read Assembly

Having obtained short reads from WGS of isolate DNA, reads are generally mapped to a reference and/or assembled *de novo* using a de Bruijn graph assembler (Zerbino and Birney, 2008; Compeau et al., 2011) (for detailed workflows see Edwards and Holt, 2013; Lynch et al., 2016). Reference-based read mapping is a fast and accurate method to characterize SNPs and detect loci of interest (Li and Durbin, 2009). Identified core genome SNPs can be used to construct strain phylogenies. In addition, rapid epidemiological surveillance of replicons and resistance genes can potentially be achieved with read mapping tools such as SRST2 (Inouye et al., 2014). However, the read mapping approach is limited when structural information is of interest: is a detected resistance gene located on chromosome or plasmid, and if the latter, which plasmid type is it associated with? One approach to overcome this is *de novo* read assembly, which can be less sensitive for SNP or locus detection (Inouye et al., 2014), but provides reference-free structural information, and can identify loci not represented on available references. **Table 2** summarizes key *in silico* tools for plasmid analysis.

**Table 2:** Summary of common *in silico* tools used for plasmid analysis.

Goal	Tool(s); reference(s)	Comments
Detect loci of interest from reads	SRST2 (Inouye et al., 2014)	Reads are mapped to a reference database using bowtie2 (Langmead and Salzberg, 2012). Some databases are included as part of the tool (e.g., PlasmidFinder, ResFinder, ARG-ANNOT) but custom databases can also be used.
Detect resistance genes from k-mers	KmerResistance (Clausen et al., 2016)	Identifies resistance genes from WGS data by examining co-occurrence of k-mers (DNA substrings of length k) between the query WGS data and a reference database of resistance genes.
Comparative plasmid genomics	ACT; BRIG (Carver et al., 2005; Alikhan et al., 2011)	Tools such as ACT and BRIG can be used to order contigs against a reference plasmid using BLAST, allowing homologies and gene content similarity to be visualized.
Detect replicon type/subtype from contigs	PlasmidFinder; pMLST (Carattoli et al., 2014)	See **Table 1**.
Detect resistance genes from contigs	ResFinder (Zankari et al., 2012)	Contigs are BLAST searched against a database of horizontally acquired resistance genes; resistance-conferring mutations are not accounted for.
	CARD (McArthur et al., 2013)	Contigs are BLAST searched against the CARD database; resistance genes are associated with an ontology allowing resistance gene metadata to be retrieved. CARD also provides the Resistance Gene Identifier tool for resistance prediction.
	ARG-ANNOT (Gupta et al., 2014)	BLAST-based tool for detection of resistance genes and resistance mutations.
Localize specific genes of interest from a contig assembly	Bandage (Wick et al., 2015)	Assembly graph visualization and annotation tool (can be used for manual repeat resolution).
	ISMapper (Hawkey et al., 2015)	Mapping-based tool which uses paired-end sequencing data to localize insertion sequences. Can be used for localizing a particular resistance locus, given a known association with a specific insertion sequence.
Distinguish plasmid from chromosomal sequences	cBar (Zhou and Xu, 2010)	Plasmid and chromosomal sequences are distinguished based on pentamer frequencies.
	Other tools	Tools such as plasmidSPAdes and PlasmidFinder may also be used to distinguish plasmid and chromosomal sequences (Arredondo-Alonso et al., 2016).
Resolve plasmid structures from ambiguous assembly graphs	PLACNET (Lanza et al., 2014)	An input assembly graph is reconfigured according to the homology of contigs to reference sequences; the assembly graph can be visualized to allow manual pruning and correction.
	Recycler (Rozov et al., 2016)	Cycles in an assembly graph are identified and sequentially extracted from the graph, favoring cycles with minimal coverage variation across constituent contigs. Assuming different genetic units have distinct copy numbers, retrieved cycles should represent individual circular elements (plasmids, circular phages). Information from paired-end reads is used to exclude cycles that do not correspond to a single circular element, but arise from repeat elements shared across different molecules.
	plasmidSPAdes (Antipov et al., 2016)	Median coverage of longer contigs is calculated to estimate chromosomal coverage; this estimate is used as a basis for filtering putative chromosomal contigs from the assembly graph. Connected components within the filtered graph are reported as putative plasmids. This approach assumes that chromosomal contig coverage differs from plasmid contig coverage.

*The tools presented here are not necessarily exhaustive and not all are intended only for plasmid analysis.*

### Determining the Genetic Context of Resistance Genes from *De novo* Assemblies

Sometimes, short reads can be assembled into complete plasmid structures, and plasmid-localized resistance genes can be identified using a combination of in silico plasmid typing and resistance gene typing methods (e.g., PlasmidFinder and ResFinder). However, complete plasmid assembly is frequently not possible (Arredondo-Alonso et al., 2016). Notably, the presence of multiple copies of the same repeat structure – a common situation in plasmid genomes – introduces assembly ambiguity, which can fragment assemblies (Pevzner et al., 2001; Treangen and Salzberg, 2011). Paired-end sequencing data can resolve repeat location, but only if the paired reads span the length of the repeat. If contigs contain sufficient informative sequence, plasmid and chromosomal contigs can be distinguished by BLAST searching against the Genbank nucleotide database (Seni et al., 2016).

Resistance genes are frequently flanked by repetitive mobile elements, and are therefore prone to poor assembly, obscuring their genetic context. There are several tools which can help resolve the location of specific loci of interest (Holt, 2015). Bandage allows visualization and annotation of the assembly graph; for example, users can zoom to unresolved repeat regions and BLAST search connecting contigs (Wick et al., 2015). If all connecting contigs match either plasmid or chromosomal references, then an ambiguously linked region can be assigned accordingly. For example, Bandage helped to reveal diverse plasmid contexts for the *mcr-1* colistin resistance gene in UK clinical isolates (Doumith et al., 2016). However, this manual approach is unfeasible when analyzing large datasets. In some cases, the genetic context of resistance genes may be inferred for large datasets using the ISMapper tool, as demonstrated by a study of 1832 isolates belonging to the successful H58 *Salmonella* Typhi lineage (Wong et al., 2015).

### Reference-Based Mapping to Track Plasmid Transmission during Short-term Outbreaks

As well as detecting loci of interest, reference-based mapping has been used to track plasmids during short-term outbreaks. Specifically, ‘index’ plasmids of an outbreak are fully assembled, often through long-read sequencing. Short reads or contigs from subsequent isolates are then mapped to the index plasmid, which is deemed present if homology is demonstrated across a given length of the reference sequence (Mathers et al., 2015; Pecora et al., 2015; Stoesser et al., 2015b). This approach implicitly assumes that the index plasmid is important throughout the study period, and that plasmid structures are relatively conserved in the short-term. In some cases, these assumptions may hold (Stoesser et al., 2014), but other studies show major structural changes can occur, including recombination of large segments (Conlan et al., 2016) as well as mobilization of resistance genes (Sheppard et al., 2016). Crucially, Sheppard et al. (2016) demonstrated that reference-based mapping can be misleading if plasmid plasticity is high. Specifically, a reference *bla*_KPC_ resistance plasmid from the index isolate was detected across diverse strains by a contig-alignment approach, leading to the initial interpretation that resistance had spread via the original *bla*_KPC_ plasmid. Instead, long-read sequencing showed that often the *bla*_KPC_ gene was actually present on a co-resident plasmid, suggesting that mobilization of *bla*_KPC_ had recruited diverse plasmids to the outbreak. It was usually not possible to determine the genetic context of *bla*_KPC_ without long reads due to long repetitive flanking sequences (Sheppard et al., 2016).

### Algorithms to Improve Plasmid Reconstruction from Fragmented Assemblies

Approaches described so far aim to address specific questions, such as the location of a resistance gene, or the short-term transmission of particular plasmids. It is yet more challenging to generate complete structures of diverse plasmids from fragmented assemblies, although several algorithms attempt to do this. The Plasmid Constellation Network (PLACNET) method (Lanza et al., 2014) takes the assembly graph and adds reference genomes as nodes; references are linked to homologous contigs, and the assembly graph is reconfigured according to the additional links. Finally, manual reconfiguration is required to retrieve disjoint connected components that should represent distinct genetic units (chromosome/plasmid), with plasmids identified by presence of replication or relaxase proteins. However, poor assembly of repetitive sequences remains a challenge for resistance gene localization, and reliance on reference sequences to order the network can lead to large-scale errors in structure. For example, de Been et al. (2014) used long-read sequencing to validate PLACNET reconstructions; in one case, plasmid contigs had been incorrectly reconstructed as two distinct plasmids rather than one, probably because the plasmid was a fusion of two previously-observed reference plasmids.

Alternative algorithms such as Recycler (Rozov et al., 2016) and plasmidSPAdes (Antipov et al., 2016) are entirely automated, and independent of reference sequences. Instead, read coverage is used to reconstruct plasmids, with the assumption that contigs from the same genetic unit should share similar coverage. However, read coverage will not distinguish different plasmids if they maintain similar copy numbers within the same host cell, nor distinguish chromosomal from plasmid contigs if a plasmid is maintained at a copy number of one across sampled cells. A recent assessment shows that Recycler and plasmidSPAdes fail to accurately reconstruct all plasmid structures from short-read WGS datasets, though the goal of identifying plasmid-derived sequence (regardless of structural accuracy) is more attainable (Arredondo-Alonso et al., 2016).

### Long-Read Sequencing

Long-read sequencing technologies, notably single molecule real-time sequencing (SMRT, Pacific Biosciences) and nanopore sequencing (Oxford Nanopore) promise to revolutionize plasmid analysis (Chin et al., 2013; Loman et al., 2015). The accurate plasmid structures generated (Ashton et al., 2014) allow for a detailed picture of plasmid epidemiology and evolution (Conlan et al., 2014; Johnson et al., 2016). However, current cost considerations have so far restricted analyses to small isolate collections. Conlan et al. (2016) showed how analysis of short-read data can guide economical use of SMRT sequencing: to examine plasmids during an outbreak, short reads were mapped to a reference, whilst unmapped reference regions, PCR amplification of marker genes, and excess reads were used as indicators of structural change, justifying investigation with long-read sequencing. However, minor structural variation (which may include resistance gene mobilization) is unlikely to be detected by this approach. Hybrid (short/long-read) assembly, using reduced long-read coverage, may help to partially mitigate current costs (Koren and Phillippy, 2015).

## Networks for Plasmid Analysis

Current plasmid typing schemes exploit a relatively small number of loci thought to best reflect the vertical (tree-like) component of plasmid evolution. However, plasmid genomes tend not to conform to tree-like evolution: co-integration events and genetic exchanges amongst plasmids mean that different parts of a plasmid genome may have different evolutionary origins (Bapteste et al., 2009). A complementary way to assess plasmid diversity involves ordering plasmids into dendrograms (de Been et al., 2014) or networks according to their gene content similarity, irrespective of whether gene-sharing stems from vertical inheritance or horizontal acquisition. In a gene-sharing network, plasmids are nodes that are linked to other plasmids if they share genes at a given sequence identity threshold (Brilli et al., 2008; Corel et al., 2015). The network topology can identify plasmids with interesting gene-sharing patterns. For example, some plasmids may act as ‘bridges’ in the gene-sharing network, straddling different groups that respectively share few genes (in graph theory terms, these plasmids have high ‘betweenness centrality’). Such plasmids may represent co-integrate plasmids, or may be important in shuttling genes across the network (Halary et al., 2010).

Making well-supported inferences from gene-sharing networks is challenging since gene-sharing might result from vertical inheritance, horizontal acquisition, or acquisition from a source not represented within the network. However, there are various ways to infer HGT events (Fondi and Fani, 2010; Tamminen et al., 2012; Fondi et al., 2016). For example, finding genes with very high sequence identity from plasmids that otherwise share few genes has been used to identify putative recent HGT events (Yamashita et al., 2014). Overall, networks are a powerful complementary tool for visualizing relationships across diverse plasmids, and generating hypotheses about the horizontal component of plasmid evolution. Gene-sharing networks only require assembly of genes, not complete plasmids, so are suited to analysis of fragmented assemblies. Since network topology is determined by constituent nodes, large unbiased plasmid datasets will produce the most informative network analyses.

## Future Prospects: WGS and Beyond

In future, optical mapping of intact plasmids could complement sequencing-based analysis. Specifically, AT-rich plasmid sequence can be fluorescently labeled, after which plasmids are elongated within nanofluidic channels and visualized with fluorescence microscopy. This allows a course-grained optical ‘barcode’ to be obtained. Optical plasmid barcoding could be used for classifying plasmids, and the course-grained structural information from the barcodes could guide plasmid assembly (Nyberg et al., 2016).

Meanwhile, current algorithms fail to accurately reconstruct all plasmids from short-read WGS datasets. Long-read sequencing will be increasingly used to determine accurate plasmid structures. Use of replicon and MOB typing for plasmid taxonomy will probably continue, but future developments could include methods to incorporate plasmid structural variation into a phylogenetic framework. Networks are a powerful tool for assessing plasmid relationships, from a functional rather than a phylogenetic perspective. Perhaps future plasmid databases could be structured as networks; when novel plasmids are added, information about their potential importance (e.g., centrality within the network) could be determined, and putative HGT events could be continually inferred. Future advances in plasmid metagenomics will enhance our knowledge of plasmids across a range of environments, and improve understanding of resistance gene reservoirs.

## Author Contributions

AO undertook literature searching, and wrote a draft manuscript. AO, HP, AS, NS, AW, MA, ME, MD, and NW suggested/implemented revisions. HP, AS, NS, AW, MA, and MD helped in planning the manuscript. AO, NS, MA, MD, ME, TP, DC, NW, AW, HP, and AS read and approved the manuscript.

## Conflict of Interest Statement

The authors declare that the research was conducted in the absence of any commercial or financial relationships that could be construed as a potential conflict of interest.
